# Supplementary material for: Eukaryotic initiation factor EIF-3.G augments mRNA translation efficiency to regulate neuronal activity
Source: eLife. 2021 Jul 29;10:e68336. doi: 10.7554/eLife.68336 (PMC8354637; doi:10.7554/eLife.68336)
Supplement: Supplementary file 1. [file elife-68336-supp1.docx]

**Supplementary File 1: Strains used in this study.**

| strain | genotype | transgene/allele description | observed results |
| --- | --- | --- | --- |
| N2 | *+* | *RRID:CGC_N2* |  |
| MT6241 | *acr-2*(*n2420*) X | G925A, V309M, also see Supplemental Table S2 |  |
| CZ22197 | *eif-3.G*(*ju807*) II | G722A, C130Y, also see Supplemental Table S2 |  |
| CZ28494 | *eif-3.G*(*ju1840*) II | G713A C714T, C127Y |  |
| CZ21759 | *eif-3.G*(*ju807*) II ; *acr-2*(*n2420*) X |  |  |
| CZ28495 | *eif-3.G*(*ju1840*) II ; *acr-2*(*n2420*) X |  |  |
| CZ22974 | *eif-3.G*(*ju1327*) / mnC1 II | *ju1327* is a 19bp deletion of nts 554-572, see Supplemental Table S2 |  |
| CZ22976 | *acr-2*(*n2420*) X ; *juEx7015* | Supplemental Table S3 |  |
| CZ22977 | *acr-2*(*n2420*) X ; *juEx7016* | Supplemental Table S3 |  |
| CZ23125 | *eif-3.G(ju807) II ; acr-2*(*n2420*) X ; *juEx7045* | Supplemental Table S3 |  |
| CZ23126 | *eif-3.G(ju807) II ; acr-2*(*n2420*) X; *juEx7046* | Supplemental Table S3 |  |
| CZ22980 | *eif-3.G(ju807) II ; acr-2*(*n2420*) X; *juEx7019* | Supplemental Table S3 |  |
| CZ22981 | *eif-3.G(ju807) II ; acr-2*(*n2420*) X; *juEx7020* | Supplemental Table S3 |  |
| CZ23791 | *eif-3.G(ju807) II ; acr-2*(*n2420*) X; *juEx7439* | Supplemental Table S3 |  |
| CZ23880 | *eif-3.G(ju807) II ; acr-2*(*n2420*) X; *juEx7440* | Supplemental Table S3 |  |
| CZ22982 | *eif-3.G(ju807) II ; acr-2*(*n2420*) X; *juEx7021* | Supplemental Table S3 |  |
| CZ22983 | *eif-3.G(ju807) II ; acr-2*(*n2420*) X; *juEx7022* | Supplemental Table S3 |  |
| CZ27881 | *eif-3.G(ju807) II ; acr-2*(*n2420*) X; *juEx8062* | Supplemental Table S3 |  |
| CZ27882 | *eif-3.G(ju807) II ; acr-2*(*n2420*) X; *juEx8063* | Supplemental Table S3 |  |
| CZ23310 | *eif-3.G*(*ju1327*) / mnC1 II ; *acr-2*(*n2420*) X |  |  |
| CZ25714 | *eif-3.G*(*ju807*) / *eif-3*.*G*(*ju1327*) II |  |  |
| CZ26828 | *eif-3.G*(*ju807*) / *eif-3*.*G*(*ju1327*) II; *acr-2*(*n2420*) X |  |  |
| CZ24063 | *juSi32*0 IV | Supplemental Table S3 |  |
| CZ24079 | *eif-3.G*(*ju1327*) / mnC1 II ; *juSi3*20 IV | Supplemental Table S3 |  |
| CZ24729 | *juSi32*0 IV ; *acr-2*(*n2420*) X | Supplemental Table S3 |  |
| CZ28107 | *eif-3.G(ju807) II; juSi32*0 IV ; *acr-2*(*n2420*) X |  |  |
| CZ24651 | *juSi331* IV | Supplemental Table S3 |  |
| CZ24652 | *juSi331* IV ; *acr-2*(*n2420*) X | Supplemental Table S3 |  |
| CZ28497 | *eif-3.G*(*ju1327*) / mnC1 II;  *juSi331* IV; *acr-2*(*n2420*) X |  |  |
| CZ631 | *juIs14* IV | ([Hallam et al. 2000](#_ENREF_21)) |  |
| CZ24161 | *eif-3.G*(*ju807*) II ; *juIs14* IV |  |  |
| CZ5808 | *juIs14* IV ; *acr-2*(*n2420*) X |  |  |
| CZ8905 | *eif-3.G(ju807) II ; juIs14* IV ; *acr-2*(*n2420*) X |  |  |
| KP2229 | *nuIs94* | ([Dittman and Kaplan 2006](#_ENREF_15)) |  |
| CZ24021 | *eif-3.G(ju807*) II ; *nuIs94* |  |  |
| CZ5815 | *acr-2*(*n2420*) X ; *nuIs94* |  |  |
| CZ24021 | *eif-3.G(ju807*) II ; *acr-2*(*n2420* )X ; *nuIs94* |  |  |
| CZ27434 | *eif-3.E*(*ok2607*) I / hT2 I, III ; *acr-2*(*n2420*) X | Supplemental Table S2 |  |
| CZ27433 | *eif-3.E*(*ok2607*) I / hT2 I, III ; *eif-3.G*(*ju807*) II ; *acr-2*(*n2420)* X | Supplemental Table S2 |  |
| CZ27435 | *eif-3.H*(*ok1353*) I / hT2 I, III ; *acr-2*(*n2420*) X | Supplemental Table S2 |  |
| CZ27436 | *eif-3.H*(*ok1353*) I / hT2 I, III ; *eif-3.G*(*ju807*) II ; *acr-2*(*n2420)* X | Supplemental Table S2 |  |
| CZ12338 | *oxSi3*9 IV | ([Qi et al. 2013](#_ENREF_52)) |  |
| CZ23854 | *eif-3.G(ju807)* II *; oxSi3*9 IV |  |  |
| CZ23203 | *acr-2*(*n2420*) X ; *juEx7056* | Supplemental Table S3 |  |
| CZ23204 | *acr-2*(*n2420*) X ; *juEx7057* | Supplemental Table S3 |  |
| CZ28152 | *acr-2*(*n2420*) X ;j*uEx8100* | Supplemental Table S3 |  |
| CZ28153 | *acr-2*(*n2420*) X ; *juEx8101* | Supplemental Table S3 |  |
| CZ26777 | *juEx7113* |  |  |
| CZ23304 | *acr-2*(*n2420*) X ; *juEx7114* | Supplemental Table S3 |  |
| CZ23305 | *acr-2*(*n2420*) X ; *juEx7115* | Supplemental Table S3 |  |
| CZ28066 | *acr-2*(*n2420*) X ; *juEx8095* | Supplemental Table S3 |  |
| CZ28067 | *acr-2*(*n2420*) X ; *juEx8096* | Supplemental Table S3 |  |
| CZ28057 | *acr-2*(*n2420*) X ; *juEx8087* | Supplemental Table S3 |  |
| CZ28058 | *acr-2*(*n2420*) X ; *juEx8088* | Supplemental Table S3 |  |
| CZ28064 | *acr-2*(*n2420*) X ; *juEx8089* | Supplemental Table S3 |  |
| CZ28065 | *acr-2*(*n2420*) X ; *juEx8090* | Supplemental Table S3 |  |
| OP433 | *unc-119*(*tm4063*) III ; *wgIs433[hlh-30::GFP(fosmid)]* | ([Sarov et al. 2006](#_ENREF_58)) |  |
| CZ28145 | *eif-3.g(ju807) II; unc-119*(*tm4063*) III ; *wgIs433[hlh-30::GFP(fosmid)]* |  | Expression unaltered by *eif-3.G*(*C130Y*) |
| CZ27913 | *acr-2(gf) X; unc-119*(*tm4063*) III ; *wgIs433[hlh-30::GFP(fosmid)]* |  | Expression in ACh-MNs enhanced by *acr-2(gf)* |
| CZ27914 | *eif-3.g(ju807)* II*; unc-119*(*tm4063*) III ; *acr-2*(*gf*) X; *wgIs433[hlh-30::GFP(fosmid)]* |  | Enhanced expression in ACh-MNs reduced by *eif-3.G*(*C130Y*); *acr-2(gf)* |
| MAH240 | *sqIs17[Phlh-30.a-hlh-30.a(cDNA)::GFP]* | ([Lapierre et al. 2013](#_ENREF_32)) |  |
| CZ28334 | *eif-3.g*(*ju807*) II; *sqIs17[Phlh-30.a-hlh-30.a(cDNA)::GFP]* |  | Expression unaltered by *eif-3.G*(*C130Y*) |
| CZ28212 | *acr-2*(*n2420*) X ; *sqIs17[Phlh-30.a-hlh-30.a(cDNA)::GFP]* |  | Expression unaltered by *acr-2*(*gf*) |
| CZ28218 | *eif-3.g*(*ju807*) II ; *acr-2*(*n2420*) X ; *sqIs17[Phlh-30.a-hlh-30.a(cDNA)::GFP]* |  | Expression unaltered by *eif-3.G*(*C130Y*); *acr-2*(*gf*) |
| CZ28491 | *unc-13(s69)* I; *wgIs433[hlh-30::GFP(fosmid)]* |  |  |
| CZ28492 | *unc-13(s69)* I; *acr-2*(*n2420*) X; *wgIs433[hlh-30::GFP(fosmid)]* |  | Expression unaltered by *acr-2(gf)* |
| CZ28493 | *unc-13(s69)* I; *eif-3.G*(*C130Y*); *acr-2*(*n2420*) X; *wgIs433[hlh-30::GFP(fosmid)]* |  | Expression unaltered by *eif-3.G*(*C130Y*); *acr-2(gf)* |
| CZ22459 | *juSi260[Pncs-2-ncs-2(cDNA)::GFP] ncs-2*(*tm1943*) I | Supplemental Table S2; ([Zhou et al. 2017](#_ENREF_75)) |  |
| CZ23225 | *juSi260[Pncs-2-ncs-2(cDNA)::GFP]*  *ncs-2*(*tm1943*) I ; *eif-3.g*(*ju807*) II | Supplemental Table S2 | Expression unaltered by *eif-3.G*(*C130Y*) |
| CZ22345 | *juSi260[Pncs-2-ncs-2(cDNA)::GFP]*  *ncs-2*(*tm1943*) I ; *acr-2*(*n2420*) X | Supplemental Table S2 | Expression unaltered by *acr-2(gf)* |
| CZ28110 | *juSi260[Pncs-2-ncs-2(cDNA)::GFP]*  *ncs-2*(*tm1943*) I ; *eif-3.g*(*ju807*) II ; *acr-2*(*n2420*) X | Supplemental Table S2 | Expression in VNC reduced by *eif-3.G*(*C130Y*); *acr-2(gf)* |
| CZ28213 | *juSi391[Pncs-2-5’UTR of eif-3.G-ncs-2(cDNA)::GFP]* *ncs-2*(*tm1943*) I | Supplemental Table S2 and S3 |  |
| CZ28340 | *juSi391[Pncs-2-5’UTR of eif-3.G-ncs-2(cDNA)::GFP]* *ncs-2*(*tm1943*) I ; *eif-3.g*(*ju807*) II | Supplemental Table S2 and S3 | Expression unaltered by *eif-3.G*(*C130Y*) |
| CZ28252 | *juSi391[Pncs-2-5’UTR of eif-3.G-ncs-2(cDNA)::GFP]* *ncs-2*(*tm1943*) I ; *acr-2*(*n2420*) X | Supplemental Table S2 and S3 | Expression unaltered by *acr-2(gf)* |
| CZ28253 | *juSi391[Pncs-2-5’UTR of eif-3.G-ncs-2(cDNA)::GFP]* *ncs-2*(*tm1943*) I ; *eif-3.g*(*ju807*) II ; *acr-2*(*n2420*) X | Supplemental Table S2 and S3 | Expression unaltered by *eif-3.G*(*C130Y*); *acr-2(gf)* |
| CZ28277 | *juSi392[Pncs-2-first 10AA of ncs-2-ncs-2(cDNA)::GFP]* *ncs-2*(*tm1943*) I | Supplemental Table S2 and S3 |  |
| CZ28312 | *juSi392[Pncs-2-first 10AA of ncs-2-ncs-2(cDNA)::GFP] ncs-2*(*tm1943*) I ; *eif-3.g*(*ju807*) II | Supplemental Table S2 and S3 | Expression unaltered by *eif-3.G*(*C130Y*) |
| CZ28291 | *juSi392[Pncs-2-first 10AA of ncs-2-ncs-2(cDNA)::GFP]* *ncs-2*(*tm1943*) I ; *acr-2*(*n2420*) X | Supplemental Table S2 and S3 | Expression unaltered by *acr-2(gf)* |
| CZ28292 | *juSi392[Pncs-2-first 10AA of ncs-2-ncs-2(cDNA)::GFP]* *ncs-2*(*tm1943*) I ; *eif-3.g*(*ju807*) II ; *acr-2*(*n2420*) X | Supplemental Table S2 and S3 | Expression in ACh-MNs reduced by *eif-3.G*(*C130Y*); *acr-2(gf)* |
| CZ28278 | *juSi393[Pncs-2-5’UTR of eif-3.G-first 10AA of ncs-2-ncs-2(cDNA)::GFP]* *ncs-2*(*tm1943*) I | Supplemental Table S2 and S3 |  |
| CZ28311 | *juSi393[Pncs-2-5’UTR of eif-3.G-first 10AA of ncs-2-ncs-2(cDNA)::GFP* *ncs-2*(*tm1943*) I ; *eif-3.g*(*ju807*) II | Supplemental Table S2 and S3 | Expression unaltered by *eif-3.G*(*C130Y*) |
| CZ28293 | *juSi393[Pncs-2-5’UTR of eif-3.G-first 10AA of ncs-2-ncs-2(cDNA)::GFP* *ncs-2*(*tm1943*) I ; *acr-2*(*n2420*) X | Supplemental Table S2 and S3 | Expression unaltered by *acr-2(gf)* |
| CZ28294 | *juSi393[Pncs-2-5’UTR of eif-3.G-first 10AA of ncs-2-ncs-2(cDNA)::GFP* *ncs-2*(*tm1943*) I ; *eif-3.g*(*ju807*) II ; *acr-2*(*n2420*) X | Supplemental Table S2 and S3 | Expression unaltered by *eif-3.G*(*C130Y*); *acr-2(gf)* |
| CZ9635 | *juEx2045* |  |  |
| CZ23321 | *hlh-30(tm1978) IV* |  |  |
| CZ28174 | *hlh-30(tm1978) IV; acr-2(n2420) X* |  |  |
| CZ28175 | *eif-3.G(ju807)* II*; hlh-30(tm1978) IV; acr-2(n2420) X* |  |  |
| CZ26759 | *eif-3.G*(*ju1327*) II / mnC1 II ; juSi363 IV ; *acr-2*(*n2420*) X | Supplemental Table S3 |  |
| CZ26760 | *eif-3.G*(*ju1327*) II / mnC1 II ; juSi366 IV ; *acr-2*(*n2420*) X | Supplemental Table S3 |  |
| CZ26494 | *juSi364* IV ; *acr-*2(*n2420*) X | Supplemental Table S3 |  |
| CZ26243 | *eif-3.G*(*ju807*)II *juSi364* IV ; *acr-*2(*n2420*) X | Supplemental Table S3 |  |
| CZ26588 | *juSi365* IV | Supplemental Table S3 |  |
| CZ26565 | *eif-3.G*(*ju807*) II ;*juSi365* IV; *acr-2*(*n2420*) X | Supplemental Table S3 |  |
| CZ26566 | *juSi365* IV ; *acr-2*(*n2420*) X | Supplemental Table S3 |  |
| CZ26656 | *juSi368* IV | Supplemental Table S3 |  |
| CZ26623 | *juSi368* IV *; acr-*2*(n2420*) | Supplemental Table S3 |  |
| CZ26480 | *eif-3.G*(*ju807*) II *; juSi368* IV ; *acr-*2(*n242*0) | Supplemental Table S3 |  |
| OP506 | *wgIs506[xbp-1::GFP(fosmid)]* | ([Sarov et al. 2006](#_ENREF_58)) | Expression not observed in ACh-MNs |
| CZ27926 | *acr-2(n2420)* X; *wgIs506[xbp-1::GFP(fosmid)]* |  | Expression not observed in ACh-MNs |
| CZ27927 | *eif-3.G(ju807)* II; *acr-2(n2420)* X; *wgIs506[xbp-1::GFP(fosmid)]* |  | Expression not observed in ACh-MNs |
| OD2955 | *dhc-1::GFP(it45)* I | ([Wang et al. 2017](#_ENREF_71)) |  |
| CZ27858 | *dhc-1::GFP(it45)* I; *acr-2*(*n2420*) X |  | Expression unaltered by *acr-2*(*gf*) |
| CZ27859 | *dhc-1::GFP(it45)* I; *eif-3.G*(*ju807*) II; *acr-2*(*n2420*) X |  | Expression unaltered by *eif-3.G*(*C130Y*); *acr-2*(*gf*) |
| OP432 | *wgIs432[zip-2::GFP(fosmid)]* | ([Sarov et al. 2006](#_ENREF_58)) |  |
| CZ27915 | *acr-2*(*n2420*) X*; wgIs432[zip-2::GFP(fosmid)]* |  | Expression unaltered by *acr-2*(*gf*) |
| CZ28021 | *eif-3.G(ju807)* II; *acr-2*(*n2420*) X*; wgIs432[zip-2::GFP(fosmid)]* |  | Expression unaltered by *eif-3.G*(*C130Y*); *acr-2*(*gf*) |
| OP638 | *wgIs638[atf-7::GFP(fosmid)]* | ([Sarov et al. 2006](#_ENREF_58)) |  |
| CZ28108 | *unc-119(tm4063)* III*; acr-2(n2420)* X; *wgIs638[atf-7::GFP(fosmid)]* |  | Expression unaltered by *acr-2*(*gf*) |
| CZ27916 | *eif-3.G(ju807)* II; *acr-2(n2420)* X; *wgIs638[atf-7::GFP(fosmid)]* |  | Observed expression is lower in ACh-MNs compared with wild type or *acr-2*(*gf*) single mutant backgrounds |
| YL651 | *let-607(tm1423)* I; *unc-119*(*ed3*) III; *vrIs121[let-607::GFP(fosmid)]* | ([Sarov et al. 2006](#_ENREF_58)) |  |
| CZ28143 | *eif-3.G(ju807)* II*; let-607(tm1423)* I; *unc-119*(*ed3*) III; *vrIs121[let-607::GFP(fosmid)]* |  | Expression unaltered by *eif-3.G*(*C130Y*) |
| CZ28119 | *acr-2(n2420)* X*; let-607(tm1423)* I; *unc-119*(*ed3*) III; *vrIs121[let-607::GFP(fosmid)]* |  | Expression unaltered by *acr-2*(*gf*) |
| CZ28111 | *eif-3.G(ju807)* II*; acr-2(n2420) X; let-607(tm1423)* I; *unc-119*(*ed3*) III; *vrIs121[let-607::GFP(fosmid)]* |  | Observed expression is lower in ACh-MNs compared with wild type or *acr-2*(*gf*) single mutant backgrounds |
| EE86 | MUP-4::GFP(juIs172) |  | Expression not observed in ACh-MNs |
| PS4263 | egl-30(md186) I; dpy-20(e1282ts) IV; syIs105(EGL-30::GFP) |  | Expression not observed in ACh-MNs |
| CZ27420 | *Pflp12*::*flp-12*::SL2::mKate2 (juEx7964) | ([McCulloch et al. 2020](#_ENREF_38)) | Expression observed in ACh-MNs |
| CZ27217 | *acr-2*(*gf*) X*; Pflp12*::*flp-12*::SL2::mKate2 (juEx7964) | ([McCulloch et al. 2020](#_ENREF_38)) | Expression in ACh-MNs unaltered by *acr-2*(*gf*) |
| CZ28109 | *eif-3.G*(*C130Y*) II; *acr-2*(*gf*) X*; Pflp12*::*flp-12*::SL2::mKate2 (juEx7964) |  | Expression unaltered by *eif-3.G*(*C130Y*); *acr-2*(*gf*) |
|  |  |  |  |
|  |  |  |  |
|  |  |  |  |
|  |  |  |  |
