# Supplementary material for: Eukaryotic initiation factor EIF-3.G augments mRNA translation efficiency to regulate neuronal activity
Source: eLife. 2021 Jul 29;10:e68336. doi: 10.7554/eLife.68336 (PMC8354637; doi:10.7554/eLife.68336)
Supplement: Supplementary file 2. [file elife-68336-supp2.docx]

**Supplementary File 2: Genotyping primers used in this study.**

| allele | primer | primer sequence (5′-3′) | Genotype* |
| --- | --- | --- | --- |
| *acr-2(n2420)* | YJ10882 | GGAATATGGGACGTGATTGGTAA | CGCTCTTGTTATGTTCTTGTT |
|  | YJ10883 | ATTATTTCTCTATTGACCGTGGTCC |  |
|  | YJ8874 | GGTATCCTTTCTGGTCGTTTCATC (sequencing primer) |  |
| *eif-3.G(ju807)* | YJ12592 | CCACACATCCAGGAAGGAGCT | TGCCGTCATTACAAAGGAAAC |
|  | YJ12593 | AACGGATTAGTTGCTCGGACGTG |  |
|  | YJ12595 | ATCATTGAGTTCTGCAATTGCACG (sequencing primer) |  |
| *eif-3.G(ju1327)* | YJ12594 | CACAACTTACGTGGCTGAAGAAG | WT- 159 bp *ju1327*- 140 bp |
|  | YJ12596 | GGACAATGAGTACTCCAATGGTCG |  |
| *ife-1(bn127)* | YJ12400 | GTTCCACGTGCCCATTTTTATTTGC | WT- 899 bp *bn127*- 309 bp |
|  | YJ12401 | GCCTGGAAACCACAAGGCAATATAATAC |  |
| *ife-2(ok306)* | YJ12402 | CTACAGCAAAGTTGAGGGCTTGAG | WT- 1,291 bp *ok306*- 879 bp |
|  | YJ12403 | GCTTGAAAATTAATTAGACAAG |  |
|  | YJ12404 | GCTGATGACGGGGCAAATCTGAG |  |
| *ife-4(tm684)* | YJ12597 | TCCAGAAATGTGCGACAAAGCTG | WT- 1,220 bp *tm684*- 429 bp |
|  | YJ12598 | ATGGAACGAATTAAAGCGGTCGAC |  |
| *lgc-46(ju825)* | YJ12599 | CACGTACTTCCACGTACAAACTCATTAG | TACTTGCAATAACTTTCCAGT |
|  | YJ12600 | GATTTACCCGCGAAGTTATGCG (sequencing primer) |  |
| *eif-3.E(ok2607)* | YJ12396 | ACAATGGAAAACCTATTCCGGC | WT- 1,222 bp *ok2607*- 644 bp |
|  | YJ12397 | CGCCTTGACTTTGTCTTGACG |  |
| *eif-3.H(ok1353)* | YJ12398 | TACAATTGACGCGCAGTTGC | WT- 1,976 bp *ok1353*- 639 bp |
|  | YJ12601 | ACCAGAAAGGAATTTCATACGGC |  |
| Si chromosome I (*juSi260, juSi391, juSi392, and juSi393*) | YJ10686 | TTTTTCAGAAATATATGCCGAGGATGTTC | WT- 623 bp with insertion- 820 bp |
|  | YJ10507 | tgtcgaccgctagtgtagcttac |  |
|  | YJ10508 | cgtctctccacgatttacacactatttg |  |
| Si chromosome IV( *juSi320, juSi331, juSi364, juSi365, juSi366, and juSi368*) | YJ10503 | GGAACAAAGGAGTTCAGATCCTGTG | WT- 562 bp with insertion- 744 bp |
|  | YJ10504 | GGAAGACCCTTAGTTCCAAACAAGTG |  |
|  | YJ11707 | CCAGACTCGACTGAATGAACAGG |  |

***** indicates deletion/insertion alleles or nucleotide changes (red) in corresponding alleles.
