# Supplementary material for: Eukaryotic initiation factor EIF-3.G augments mRNA translation efficiency to regulate neuronal activity
Source: eLife. 2021 Jul 29;10:e68336. doi: 10.7554/eLife.68336 (PMC8354637; doi:10.7554/eLife.68336)
Supplement: Supplementary file 3. [file elife-68336-supp3.docx]

**Supplementary File 3: Constructs and related primers used in this study.**

| **construct #** | **transgene(s)** | **primer** | **sequence (5' to 3')** |
| --- | --- | --- | --- |
| pCZGY3006, pCZGY3007 | *juEx7015/juEx7016, juEx7045/juEx7046* | YJ11959 | AGGGATTACAATCAACGAAAGG |
|  |  | YJ11960 | AAGTAGTCTTTCCAGCGTTGTC |
| pCZGY3008, pCZGY3009 | *juEx7045/juEx7046, juEx7019/juEx7020, juEx7439/juEx7440, juEx7021/juEx7022, juEx8062/juEx8063* | YJ74 | GTTTAATTACCCAAGTTTGAG |
|  |  | YJ11560 | AACTATGATATTTTACATTGGACAG |
| pCZGY3018, pCZGY3019 | *juSi320/juSi321* | YJ12604 | GGTGGAGGTGGAGCAACTGGCATTAACGCAATG |
|  |  | YJ12605 | AGTTCTTCTCCTTTACTCATAGCTGTAACTTTACTACCTC |
|  |  | YJ12602 | CTATGAGTAAAGGAGAAGAACTTTTCACTGGAGTTGTCC |
|  |  | YJ12603 | TGCTCCACCTCCACCTCCTTTGTATAGTTCATCCATGCC |
| pCZGY3508 | *juEx8087/juEx8088* | YJ12453 | tcaggaggacccttggctagcgtcgacggtacatcatgcgtccactttcgctga |
|  |  | YJ12454 | ggccgatgcggagctcagataattttaactcaaaataatatttatttatgaaaaaaaaaacaattaaaagccacc |
| pCZGY3509 | *juEx8089/juEx8090* | YJ12457 | agagacgcgatgcaggtcacacagacgtctgtctctgc |
|  |  | YJ12458 | acctgcatcgcgtctcttcctcctccaaggcaaatgtg |
| pCZGY3512 | *juEx8095/juEx8096* | YJ12463 | TAAaagtgcagagccagtcagg |
|  |  | YJ12464 | ctggctctgcacttTTAgtcgtc |
|  |  | YJ12465 | GCTGCCGCTgttacatttgaatcaagagatgacgccgcc |
|  |  | YJ12466 | tcaaatgtaacAGCGGCAGCtcctttcgggagaccagt |
| pCZGY3026, pCZGY3027 | *juEx7113, juEx7114/juEx7115,* | YJ11561 | ATCTTCCGTAAGAGATGAACGAAGACGAACTTCGTG |
|  |  | YJ11562 | GTTCATCTCTTACGGAAGATTGGTGACACGGCATGT |
| pCZGY3536, pCZGY3537 | *juSi363,  juSi366* | YJ12607 | CTAGAAAGTATAGGAACTTCGCATGAGGGATTACAATCAACGAAAGGAAT |
|  |  | YJ12608 | GTCATCGTCTTTATAATCcatAGCTGTAACTTTACTACCTGAAAAAC |
|  |  | YJ12419 | GGTAGTAAAGTTACAGCTatgGATTATAAAGACGATGACGATAAG |
|  |  | YJ12420 | AGTaggatgagacagcTTAGTTGCTCGGACGTGTCC |
|  |  | YJ12421 | CGtccgagcaactaaGCTGTCTCATCCTACTTTCACC |
|  |  | YJ12422 | CAATTCTTctcctttactcatGATGCGTTGAAGCAGTTTCCC |
|  |  | YJ12423 | CTgcttcaacgcatcATGAGTAAAGGAGAAGAATTGTTCAC |
|  |  | YJ12424 | gcaaaaatcaatagaggaccgctacacaTTACTTGTAGAGCTCGTCC |
| pCZGY3538, pCZGY3539, pCZGY3540 | *juSi364,  juSi365,  juSi368* | YJ12164 | aagtataggaacttcgcatgAAGCTTTTGGTTTTCACAATTTTC |
|  |  | YJ12418 | gtcatcgtctttataatccatTTGAACAAGAGATGCGGAAAATAG |
|  |  | YJ12419 | ccgcatctcttgttcaaATGGATTATAAAGACGATGACGATAAG |
|  |  | YJ12420 | AGTaggatgagacagcTTAGTTGCTCGGACGTGTCC |
|  |  | YJ12421 | CGtccgagcaactaaGCTGTCTCATCCTACTTTCACC |
|  |  | YJ12422 | CAATTCTTctcctttactcatGATGCGTTGAAGCAGTTTCCC |
|  |  | YJ12423 | CTgcttcaacgcatcATGAGTAAAGGAGAAGAATTGTTCAC |
|  |  | YJ12424 | gcaaaaatcaatagaggaccgctacacaTTACTTGTAGAGCTCGTCC |
| pCZGY3526 | *juSi391* | YJ12554 | tcaaagaaatcgccgacttaaactacacggaggcttcaacaatc |
|  |  | YJ12555 | tcaaacttgggtaattaaacctgcaaaaaaacgagaaattcatgtaacaaaac |
|  |  | YJ12556 | gtttaattacccaagtttgaggtagtaaagttacagctatgggaatcaagggttccaagc |
|  |  | YJ12557 | aagtataggaacttcgcatgaagtcaagagaggtggaacgagag |
| pCZGY3533 | *juSi392* | YJ12554 | tcaaagaaatcgccgacttaaactacacggaggcttcaacaatc |
|  |  | YJ12579 | TCCGCCGCCACCTGCCTTGATTCCCATggtttgggc |
|  |  | YJ12580 | TCAAGGCAGGTGGCGGCGGATCCatggtgagcaagggc |
|  |  | YJ12557 | aagtataggaacttcgcatgaagtcaagagaggtggaacgagag |
| pCZGY3534 | *juSi393* | YJ12554 | tcaaagaaatcgccgacttaaactacacggaggcttcaacaatc |
|  |  | YJ12555 | tcaaacttgggtaattaaacctgcaaaaaaacgagaaattcatgtaacaaaac |
|  |  | YJ12581 | aattacccaagtttgaggtagtaaagttacagctATGGGAATCAAGGCAGGTGGCGGCGG |
|  |  | YJ12557 | aagtataggaacttcgcatgaagtcaagagaggtggaacgagag |
