# Supplementary material for: Eukaryotic initiation factor EIF-3.G augments mRNA translation efficiency to regulate neuronal activity
Source: eLife. 2021 Jul 29;10:e68336. doi: 10.7554/eLife.68336 (PMC8354637; doi:10.7554/eLife.68336)
Supplement: Supplementary file 4. [file elife-68336-supp4.docx]

**Supplementary File 4:** **Number of mapped reads in seCLIP replicate datasets obtained after sequencing and CLIPPER filtering.**

| **Sample** | **Replicate** | **Library** | **Uniquely Mapped Reads** |
| --- | --- | --- | --- |
| **IgG(-)** | **-** | **Input** | 428,682 |
|  |  | **CLIP** | 192,259 |
| **WT** | **1** | **Input** | 1,136,562 |
|  |  | **CLIP** | 1,699,202 |
|  | **2** | **Input** | 1,289,834 |
|  |  | **CLIP** | 1,572,635 |
| **C130Y** | **1** | **Input** | 3,026,636 |
|  |  | **CLIP** | 1,214,802 |
|  | **2** | **Input** | 1,671,363 |
|  |  | **CLIP** | 1,724,553 |
| **∆RRM** | **1** | **Input** | 1,551,766 |
|  |  | **CLIP** | 543,913 |
|  | **2** | **Input** | 1,310,855 |
|  |  | **CLIP** | 892,645 |
