# Supplementary material for: Eukaryotic initiation factor EIF-3.G augments mRNA translation efficiency to regulate neuronal activity
Source: eLife. 2021 Jul 29;10:e68336. doi: 10.7554/eLife.68336 (PMC8354637; doi:10.7554/eLife.68336)
Supplement: Supplementary file 5. [file elife-68336-supp5.docx]

**Supplementary File 5:** **Number of read clusters detected in each dataset after subtraction of IgG control background.**

|  | **coding** | | **non-coding** | |
| --- | --- | --- | --- | --- |
|  | **clusters** | ***genes*** | **clusters** | ***genes*** |
| **EIF-3.G(WT)** | 211 | 194 | 109 | 93 |
| **EIF-3.G(C130Y)** | 153 | 144 | 143 | 123 |
| **EIF-3.G(∆RRM)** | 525 | 454 | 156 | 141 |
