# Supplementary material for: Eukaryotic initiation factor EIF-3.G augments mRNA translation efficiency to regulate neuronal activity
Source: eLife. 2021 Jul 29;10:e68336. doi: 10.7554/eLife.68336 (PMC8354637; doi:10.7554/eLife.68336)
Supplement: Supplementary file 6. [file elife-68336-supp6.docx]

**Supplementary File 6: Number of EIF-3.G footprints detected in each dataset after subtraction of background from both IgG and ∆RRM controls.**

|  | **coding** | | **non-coding** | |
| --- | --- | --- | --- | --- |
|  | **clusters** | ***genes*** | **clusters** | ***genes*** |
| **EIF-3.G(WT)** | 211 | 194 | 109 | 93 |
| **EIF-3.G(C130Y)** | 153 | 144 | 143 | 123 |
