# Supplementary material for: Eukaryotic initiation factor EIF-3.G augments mRNA translation efficiency to regulate neuronal activity
Source: eLife. 2021 Jul 29;10:e68336. doi: 10.7554/eLife.68336 (PMC8354637; doi:10.7554/eLife.68336)
Supplement: Transparent reporting form [file elife-68336-transrepform.pdf]

eLife

Westbrook Centre
Milton Road
Cambridge CB4 1YG, UK

P 01223 855340
W elifesciences.org
T @elife

eLife's transparent reporting form

We encourage authors to provide detailed information *within their submission* to facilitate the interpretation and replication of experiments. Authors can upload supporting documentation to indicate the use of appropriate reporting guidelines for health-related research (see EQUATOR Network), life science research (see the BioSharing Information Resource), or the ARRIVE guidelines for reporting work involving animal research. Where applicable, authors should refer to any relevant reporting standards documents in this form.

If you have any questions, please consult our Journal Policies and/or contact us: editorial@elifesciences.org.

Sample-size estimation

- You should state whether an appropriate sample size was computed when the study was being designed
- You should state the statistical method of sample size computation and any required assumptions
- If no explicit power analysis was used, you should describe how you decided what sample (replicate) size (number) to use

Please outline where this information can be found within the submission (e.g., sections or figure legends), or explain why this information doesn't apply to your submission:

> For all quantification of convulsion behaviour, we used a minimum of 15 animals per strain based on previously published standards. For strains containing extrachromosomal array transgenes, we analyzed two independent transgenic lines, following the standard practice among *C. elegans* researchers.
> For quantification of GFP fluorescence intensity, a post-hoc power analysis was used to determine whether the sample size was appropriate.
> All sample sizes are reported on the corresponding graphs or figure legends.

Replicates

- You should report how often each experiment was performed
- You should include a definition of biological versus technical replication
- The data obtained should be provided and sufficient information should be provided to indicate the number of independent biological and/or technical replicates
- If you encountered any outliers, you should describe how these were handled
- Criteria for exclusion/inclusion of data should be clearly stated
- High-throughput sequence data should be uploaded before submission, with a private link for reviewers provided (these are available from both GEO and ArrayExpress)

Please outline where this information can be found within the submission (e.g., sections or figure legends), or explain why this information doesn't apply to your submission:

eLife Sciences Publications, Ltd is a limited liability non-profit non-stock corporation incorporated in the State of Delaware, USA, with company number 5030732, and is registered in the UK with company number FC030576 and branch number BR015634 at the address Westbrook Centre, Milton Road, Cambridge CB4 1YG.

Qualitative observations of convulsion behaviour and fluorescence intensity were made > 3 times during the process of strain construction. Quantification was made from at least two independent data sets, with genotype generally blinded to observers. For extrachromosomal array containing animals, an equivalent number of animals from two replicate strains was used.

We used two biological replicates for all seCLIP experiments and one IgG negative control (seCLIP performed in a wild type background). In our seCLIP experiments, the biological replicates were animals of the same strain grown on different media plates under identical conditions.

No outliers were excluded from any experiment. Details of all data analysis are described in the Methods of the submitted manuscript.

Raw and analyzed seCLIP datasets from this study have been uploaded to the Gene Expression Omnibus (GEO) under accession number GSE152704.

eLife Sciences Publications, Ltd is a limited liability non-profit non-stock corporation incorporated in the State of Delaware, USA, with company number 5030732, and is registered in the UK with company number FC030576 and branch number BR015634 at the address Westbrook Centre, Milton Road, Cambridge CB4 1YG.

Statistical reporting

- Statistical analysis methods should be described and justified
- Raw data should be presented in figures whenever informative to do so (typically when N per group is less than 10)
- For each experiment, you should identify the statistical tests used, exact values of N, definitions of center, methods of multiple test correction, and dispersion and precision measures (e.g., mean, median, SD, SEM, confidence intervals; and, for the major substantive results, a measure of effect size (e.g., Pearson's r, Cohen's d)
- Report exact p-values wherever possible alongside the summary statistics and 95% confidence intervals. These should be reported for all key questions and not only when the p-value is less than 0.05.

Please outline where this information can be found within the submission (e.g., sections or figure legends), or explain why this information doesn't apply to your submission:

> Statistical analyses are described in the figure legends and methods section. All sample sizes, data presentation as SEM or SD, p-values and r-values are reported within the corresponding figure or indicated within the figure legends.

(For large datasets, or papers with a very large number of statistical tests, you may upload a single table file with tests, Ns, etc., with reference to sections in the manuscript.)

Group allocation

- Indicate how samples were allocated into experimental groups (in the case of clinical studies, please specify allocation to treatment method); if randomization was used, please also state if restricted randomization was applied
- Indicate if masking was used during group allocation, data collection and/or data analysis

Please outline where this information can be found within the submission (e.g., sections or figure legends), or explain why this information doesn't apply to your submission:

> Experimental groups were established based on animal genotype, including controls. Animal behaviour and imaging was scored with genotype blinding to observer, when possible. Relevant information is described in the methods section.

Additional data files ("source data")

- We encourage you to upload relevant additional data files, such as numerical data that are represented as a graph in a figure, or as a summary table
- Where provided, these should be in the most useful format, and they can be uploaded as "Source data" files linked to a main figure or table
- Include model definition files including the full list of parameters used
- Include code used for data analysis (e.g., R, MatLab)
- Avoid stating that data files are "available upon request"

Please indicate the figures or tables for which source data files have been provided:

> Source data as an excel file with spreadsheet tabs for each figure data is uploaded.
